# Inhibition of acid sphingomyelinase activity ameliorates endothelial dysfunction in *db/db* mice

**DOI:** 10.1042/BSR20182144

**Published:** 2019-04-23

**Authors:** Meng Jiang, Shanya Huang, Wang Duan, Qiaoshu Liu, Minxiang Lei

**Affiliations:** Xiangya Hospital of Central South University, Hunan Province, Xiangya Street 78, Changsha 410008, China

**Keywords:** acid sphingomyelinase, ceramide, diabetes, endothelial dysfunction

## Abstract

Acid sphingomyelinase (aSMase) plays an important role in endothelial dysfunction. Here, we show that elevated aSMase activity and ceramide content were reduced by desipramine treatment in diabetic animals. The inhibitor of aSMase, desipramine, improved vascular dysfunction in db/db mice. High glucose (HG)-induced up-regulation of aSMase activity and ceramide levels were restored by treatment with aSMase siRNA or desipramine in endothelial cells. In addition, aSMase siRNA or desipramine treatment increased the release of nitric oxide (NO) and the phosphorylation of endothelial NO synthase (eNOS) in diabetic mouse aortas and aortic endothelial cells with HG.

These results indicate that inhibition of aSMase/ceramide pathway improves endothelium-dependent vascular relaxation (EDR) largely through regulating the eNOS/NO pathway in diabetic animals.

## Introduction

Diabetes includes a group of chronic diseases characterized by hyperglycemia and/or hyperinsulinemia. Vascular complications are the most serious manifestations of these diseases and the leading cause of death in diabetic patients.

Sphingolipids, especially ceramide, have been reported to play an important role in the pathogenesis of diabetic vascular complications [1]. Ceramide accumulation is able to inactivate the Akt and/or IRS signaling pathway and leads to insulin resistance [2]. Inhibition of ceramide accumulation improves obesity-related metabolic disorders [3–6]. Ceramide can be generated by sphingomyelinase pathway and *de novo* pathway. The sphingomyelinase pathway is an important regulator of ceramide metabolism in diabetes [7]. Sphingomyelinase, the key enzyme in the sphingomyelinase pathway, is divided into acid, neutral, or alkaline sphingomyelinases according to their pH optima. Activation of acid sphingomyelinase (aSMase) was observed in diabetic animals and patients [8–10].

Endothelial dysfunction is well recognized as the basis of diabetic vascular complications. Nitric oxide (NO) bioavailability reduction is the main reason for endothelial dysfunction in diabetes mellitus. Previous studies reported that inhibition of ceramide accumulation improved vascular function by regulating endothelial NO synthase (eNOS) activity and NO generation [6,11–13]. Therefore, aSMase/ceramide metabolism could be a potent therapeutic target for diabetic vascular complications.

The present study investigated the hypothesis that *in vivo* and *in vitro* treatment with aSMase siRNA or desipramine improves vascular function in diabetic animals by inhibiting aSMase activity and ceramide accumulation.

## Methods and materials

### Animals

Ten 12-week-old male diabetic mice (C57BL/KsJ background) lacking the gene encoding the leptin receptor (*db/db*) and C57 mice without diabetes were purchased from the Model Animal Research Center of Nanjing University. All mice were maintained in a well-ventilated holding room (at 24 ± 1°C, at stable humidity, on a reverse 12  h/12  h light-dark cycle, with unrestricted access to water and food).

C57BL/KsJ diabetic (*db/db*) mice and wild-type C57 control mice were divided into three groups: normal control group (NC group, *n*=12), diabetes control group (DC group, *n*=12), and diabetic mice treated with desipramine group (10 mg/kg intraperitoneal injection every other day, Sigma) (DTD group, *n*=12). The NC group and the DC group were treated with an equivalent amount of solvent (0.9% NaCl). Desipramine was dissolved in 0.9% NaCl, and all injections were performed in a volume of 1 ml/100 g of body weight. The experimental protocol was approved by the ethical committee of Central South University. The investigation conformed to the Guide for the Care and Use of laboratory Animals published by the US National Institutes of Health.

### Cell culture, siRNA (small interfering RNA) transfection and treatment

Rat aortic endothelial cells (RAOECs) were obtained from Cell Applications. The cells were grown at 37°C and 5% CO2 in a humidified environment. The cells were seeded in a six-well plate and received different treatments, such as NC (normal control, 5 mM D-glucose), HG (high glucose, 25 mM D-glucose), LG (L-glucose, 25 mM L-glucose + 5 mM D-glucose), DES (desipramine, 10 μM). The desipramine concentrations used were based on our previous study [14].

For siRNA experiments, the specific aSMase (si-aSMase) and negative control siRNA (si-Con) were obtained from RiboBio Co. Ltd. (Guangzhou, China). The cells were seeded in 6-well plate and grown at about 70% confluency. Then, we transfected the siRNA to the cells using transfection kit according to the manufacturer’s protocol (RiboBio Co. Ltd. Guangzhou). After 48 h, the cells were treated with HG. The siRNA sequence was as follows:

si-aSMase-1: 5′-GCCUCAUCUCUCUCAAUAUdTdT-3′ (sense)

3′- dTdTCGGAGUAGAGAGAGUUAUA-5′ (antisense);

si-aSMase-2: 5′-CCAGUGCAACUACCUACAUdTdT-3′ (sense)

3′-dTdTGGUCACGUUGAUGGAU GUA-5′ (antisense);

si-aSMase-3: 5′-GUCUAUUCACCGCCAUCAAdTdT-3′ (sense)

3′-dTdTCAGAUAAGUGGCGGUAGUU-5′ (antisense).

### aSMase activity

An ultra performance liquid chromatography (UPLC) system was used to detect aSMase activity [15]. Briefly, 3 µl of plasma or tissue lysis was mixed with 3 µl of aSMase buffer [200 µm BODIPY-labeled C12-Sphingomyelin (Thermofisher Scientific, MA, U.S.A.)]. Then, we transferred this mixture into the assay buffer [0.2 M sodium acetate (pH = 5.0) containing 0.2 mM ZnCl2 and 0.2% Igepal CA-630] and mixed it well. Subsequently, the samples were incubated at 37°C. After 20 h, the reaction was stopped by adding 100 µl of ethanol. Finally, the product was detected and quantified using the UPLC system (Waters, MA, U.S.A.).

### Ceramide assay

The ceramide content was quantified according to a previously described method [16]. First, lipids were extracted from the plasma and the aorta. Twenty-five µl of sample was mixed with 150 µl of dichloromethane/methanol (1:2; v/v). Then, we added 100 µl of 1 M NaCl and 100 µl of dichloromethane. After centrifugation, 100 µl of the organic phase was transferred to a new tube. The organic phase was dried and dissolved it in 20 µl of 2% Igepal-CA630. Second, we detected the ceramide content. Two µl of lipid solution was mixed with 2 µl of ceramide substrate buffer and incubated at 37°C. After 1 h, this reaction solution was mixed with 56 µl of naphthalene-2,2-dicarboxaldehyde recreation buffer and incubated at 50°C for 10 min. Finally, the product was detected and quantified using the UPLC system. In the UPLC system, we used a reversed phase column (Acquity BEH Shield RP18, 2.1 × 50 mm, 1.7 µm, Waters, U.S.A.) to determine total ceramide.

### Vascular function

The vascular function was measured using a previously described method [17]. In brief, mice were anesthetized, and the aorta was isolated. We cut two vascular rings with a length of 2–3 mm from each aorta. Then, we transferred the ring to the chamber of the myograph system (Danish Myo Technology, Winston-Salem, NC) containing oxygenated Krebs solution. The small wires were guided through the lumen of the artery and attached to a force-displacement transducer for isometric force measurements. Subsequently, arteries were equilibrated in oxygenated buffer (95% O_2_ and 5% CO_2_) at 37°C. After 30 min, the vessels were stretched to tension elicited by 1 g. The arteries were incubated for an additional 1 h, and the buffer solution was changed every 15 min. After the equilibration period, the responsiveness of each individual artery was checked by successive vasoconstriction to administration of different drugs. The stable contraction was produced by phenylephrine (1 μM) in each ring. Relaxation responses to acetylcholine (10^−8^ mol/l to 10^−6^ mol/l) were determined. Endothelium-independent relaxation was tested using sodium nitroprusside (10^−8^ mol/l to 10^−4^ mol/l).

### NO assay

NO is easily transformed into NO_2_^−^ and NO_3_^−^ *in vivo*. Hence, we determined the derivative content to index NO production. The total nitrate/nitrite concentration in the serum and the aorta was quantified by a commercial kit (Nanjing Jiancheng Bioengineering Institute) according to the manufacturer’s instructions. The absorbance was detected at 550 nm. The arterial NO content was expressed as ng/mg protein, and the serum NO content was expressed as ng/l.

*In situ* NO production was detected by using DAF-FM diacetate (molecular probes) as previously described [18]. In brief, the endothelial cells were seeded in a glass chamber and treated with different treatments. Subsequently, the cells were rinsed with normal physiological saline solution (NPSS) and incubated with 10 μM DAF-FM diacetate at 37°C for 15 min. Isolated aortas from mice were embedded in OCT compound. The frozen aortic segments were cut into 8-μm thick sections. The sections were incubated in NPSS containing 10 μM DAF-FM diacetate at 37°C for 15 min. The NO content was measured by the fluorescence intensity using confocal microscopy (Leica, German). Pictures of four different fields were taken to analysis the fluorescence intensity.

### Metabolism assays

The insulin content was measured by radioimmunoassay using commercially available radioimmunoassay kits and a radioimmuno gamma counter (Science and Technology Development CO. USTC) according to the manufacture’s instruction. Metabolism indices were measured by using the Vitalab Selectra E sequential multiple analyzers according to the manufacture’s instruction. Serum blood glucose level was measured using the glucometer meter (Abbott Laboratories, MediSense Products Inc., Bedford, MA, U.S.A.)

### Vascular pathology

The aortas were isolated and fixed with 4% paraformaldehyde. Then, the aortas were opened longitudinally and pinned down on a black plate. Lipids were stained with Oil Red O, and photographs were taken with a digital camera. Meanwhile, the aortas were fixed in 4% paraformaldehyde and embedded in paraffin. Then, 5-µm sections were cut and stained with hematoxylin and eosin (H&E) and Masson’s trichrome stain kit. Pictures of four different fields were taken by a high-power microscope and digital imaging system. The intima-media thickness was determined by Image Proplus 6.0.

In addition, the aorta was fixed in 2.5% glutaraldehyde and then postfixed in 2% osmic acid. Ultrathin sections were cut from the embedded blocks and stained with plumbi acetas and plumbi nitras. The sections were examined under a JEOL-1230 transmission electron microscope (Japan).

### Western blot analysis

Aortic tissue and endothelial cells were homogenized and lysed in RIPA buffer with protease Inhibitors (Thermofisher). Then, the lysates were centrifuged at 14500 ***g*** for 15 min, and the supernatant was collected. The protein concentration was determined by the BCA method (Beyotime Biotechnology). The protein samples were mixed with Laemmli sample buffer (Bio‐Rad Laboratories) and boiled at 95°C for 5 min. The protein samples (30 μg) loaded in each lane were separated on a 10% SDS-polyacrylamide gel. The proteins were transferred to 0.45-mm PVDF membranes (Millipore). Then, the membranes were blocked with 5% nonfat dry milk in Tris-buffered saline containing 1% Tween 20. Next, the membranes were incubated with primary antibodies, including total eNOS (9572, 1:1000), phospho-eNOS-Ser1177 (9571, 1:1000), aSMase (sc-11352, 1:400) and GAPDH (sc-47724, 1: 10000). Then, the membranes were incubated with HRP-conjugated secondary antibodies. The blots were incubated with chemiluminescent substrate (Thermo), and bands were detected by X-ray film exposure or the ChemiDoc system (Bio-Rad). The band density was analyzed by software Image J.

### Statistical analysis

All data are presented as the mean ± S.E. and analyzed by one-way ANOVA using SPSS 14.0 software. Significance was accepted at *P*<0.05.

## Results

### The effects of inhibiting aSMase/ceramide pathway on systemic metabolic homeostasis in *db/db* mice

**Figure 1:**
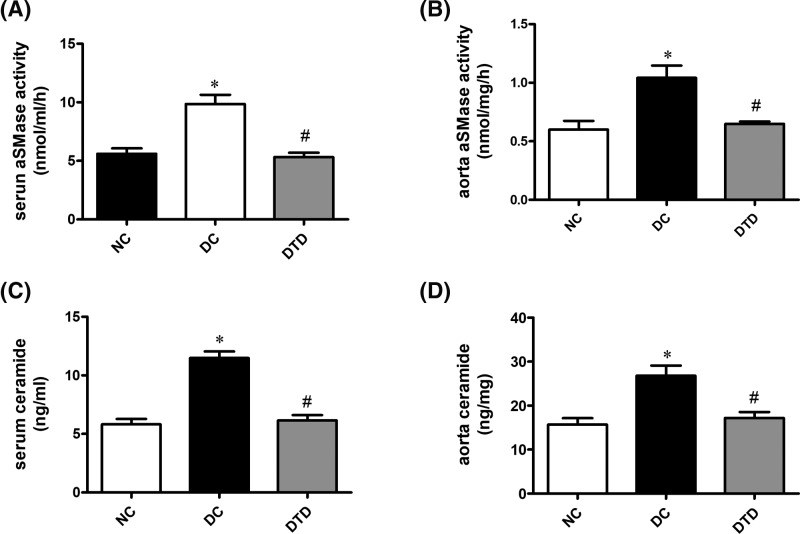
Desipramine treatment inhibits aSMase activity and ceramide accumulation in diabetic mice (**A**) Effect of desipramine on serum aSMase activity (*n*=10). (**B**) Effect of desipramine on aortic aSMase activity (*n*=10). (**C**) Effect of desipramine on serum ceramide content. (**D**) Effect of desipramine on aortic ceramide content (*n*=10). Data are shown as the mean ± S.E.M. with *n*=10 animals per group. ^*^P<0.05 compared with the NC group. ^#^*P*<0.05 compared with the DC group.

Chronic treatment with desipramine restored the up-regulated aSMase activity (Figure 1A–C) and ceramide content (Figure 1B–D). From Table. 1, the data suggested that inhibiting aSMase/ceramide pathway in diabetic mice decreased glucose and insulin levels and improved insulin resistance (Table 1). Desipramine also decreased the lipid profile in diabetic mice, but the difference was not statistically significant (Table 1).

**Table 1:** The effects of desipramine on metabolic indices

Index	NC group	DC group	DTD group
FBS (mmol/l)	8.44 ± 2.48	30.98 ± 5.63^*^	25.32 ± 2.84^*#^
TG (mmol/l)	0.80 ± 0.16	0.94 ± 0.57	0.81 ± 0.32
TC (mmol/l)	1.44 ± 0.36	3.47 ± 1.08^*^	2.87 ± 0.36^*^
HOMA-IR	2.02 ± 0.69	18.65 ± 6.37^*^	11.15 ± 3.84^*#^
Insulin (μIU/l)	7.86 ± 2.02	23.49 ± 4.78^*^	18.94 ± 4.32^*#^

All indices were determined after desipramine treatment for 12 weeks in NC group (*n*=6), DC group (*n*=6), and MTD group (*n*=6). Abbreviations, FBS, fasting blood sugar; HOMA-IR, homeostasis model assessment-estimated insulin resistance; TC, total cholesterol; TG, triglyceride. Values are means ± S.D. ^*^P<0.05, contrasted to normal control group. ^#^*P*<0.05, contrasted to diabetic control group.

### The effects of inhibiting the aSMase/ceramide pathway on endothelial dysfunction in db/db mice

**Figure 2:**
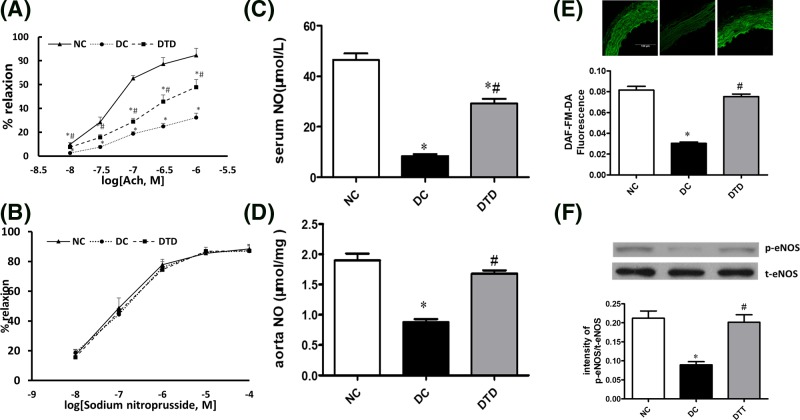
Desipramine treatment improved endothelium-dependent vascular function in diabetic mice Acetylcholine (ACh)‐induced endothelium‐dependent (**A**) and SNP‐induced endothelium‐independent (**B**) relaxations of aortic rings from db/db mice treated for 12 weeks with vehicle or desipramine. Chronic treatment with desipramine increased the level of NO in the serum (**C**) and aortas (**D**) of db/db mice. (**E**) NO generation was measured by DAF-FM-DA fluorescence under various treatments. Desipramine treatment increased the level of NO in the aortas of db/db mice. (**F**) Desipramine treatment increased the level of eNOS phosphorylation in aortas from db/db mice. Data are shown as the mean ± S.E.M. with *n*=5 animals per group. ^*^P<0.05 compared with the NC group. ^#^*P*<0.05 compared with the DC group.

Diabetes-related vascular dysfunction was partly improved by desipramine treatment (Figure 2A). However, the endothelium-independent vasorelaxation response (EIR) was normal in all animals, indicating an endothelium-specific defect (Figure 2B). In addition, treatment with desipramine restored the decreased NO release (Figure 2C–E) and phosphorylation of eNOS at Ser1177 (Figure 2F) in db/db mouse aortas. Therefore, ceramide accumulation in the vasculature likely impairs vascular function by reducing NO generation and eNOS phosphorylation.

### Morphological characteristics of mouse aortic tissue

**Figure 3:**
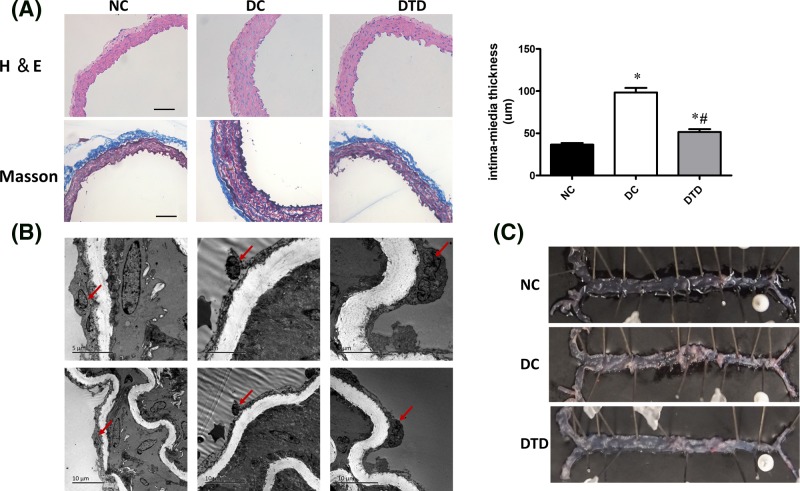
Desipramine treatment improved the morphological changes in aortas of *db/db* mice (**A**) Aortic histological sections were stained with H&E and trichrome. Desipramine treatment decreased the intima-media thickness of aortas from db/db mice. Original magnification is 200×. Scale bars represent 100 μm. (**B**) The endothelium structure changed as determined by electron microscopy observation among the three groups. Endothelial cells (bold arrow). Desipramine treatment prevented endothelial denudation of aortas from db/db mice. (**C**) Examination of the lipid deposition stained with Oil Red O. Data are shown as the mean ± S.E.M. with *n*=5 animals per group. ^*^P<0.05 compared with the NC group. ^#^*P*<0.05 compared with the DC group.

From the H&E- and Masson’s trichrome-stained tissue sections (Figure 3A), vascular remodeling and proliferation of vascular smooth muscle cells and endothelial injury were observed in the DC group compared with the control group. In addition, desipramine suppressed vascular remodeling, endothelial injury and proliferation of vascular smooth cells.

The arterial electron micrographs (Figure 3B) revealed that endothelial denudation existed in the DC group but not in the NC group or the DTD group. Therefore, the decrease in NO production might be partly caused by endothelial denudation in the DC group. As shown in Figure 3C, there were no atherosclerotic lesions among all groups, which suggested that the improvement of vascular function was independent of the atherosclerotic lesions.

### HG impairs NO production and stimulates aSMase activity in endothelial cells

**Figure 4:**
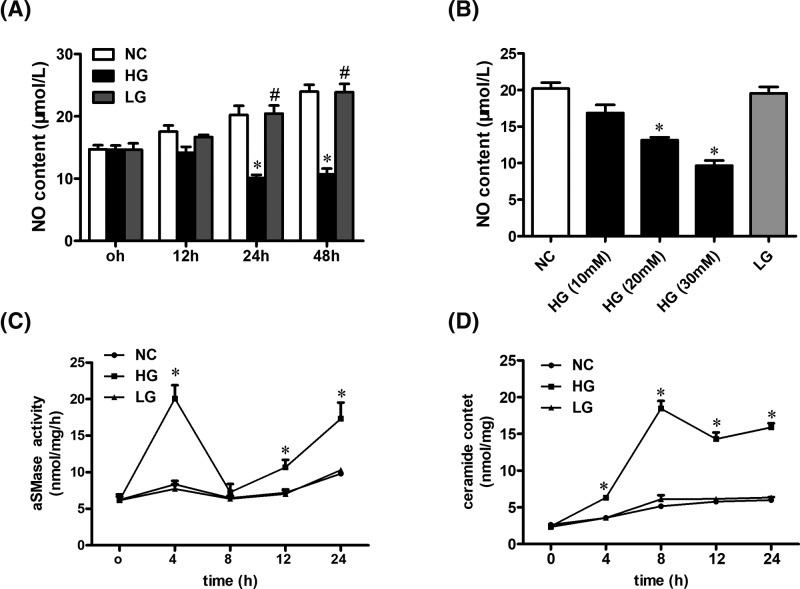
HG stimulates aSMase activity and enhances ceramide production in endothelial cells (**A**) Examination of the effect of HG or LG on NO production at different time points in endothelial cells (0, 12, 24, and 48 h). (**B**) Examination of the effect of D-glucose on NO production at different concentrations in endothelial cells (0, 12, 24, and 48 h). (**C**) Examination of the effect of HG on aSMase activity at different time points (0, 4, 8, 12, and 24 h). (**D**) Examination of the effect of HG on ceramide content at different time points (0, 4, 8, 12, and 24 h). Data are shown as the mean ± S.E.M. with *n*=4–5 per group. ^*^P<0.05 compared with the NC group. ^#^*P*<0.05 compared with the HG group.

The endothelial cells were treated with D-glucose at different time points (Figure 4A) and different concentrations (Figure 4B). The results proved that HG impaired NO release in a time- and dose-dependent pattern. Based on these findings, we used D-glucose at an optimal concentration and time (25 mM, 24 h) in subsequent experiments. In addition, the aSMase activity (Figure 4C) and ceramide content (Figure 4D) increased in endothelial cells induced by high-glucose at different time points. LG (high osmotic pressure) did not affect the aSMase activity and ceramide content (Figure 4C,D). Additionally, the aSMase activity and ceramide production were stimulated within 4-h treatment. These results suggested that the levels of aSMase-generated ceramide got increased before the NO production was inhibited.

### Inhibition of aSMase activity reverses the HG-induced impaired eNOS/NO pathway in cell culture

**Figure 5:**
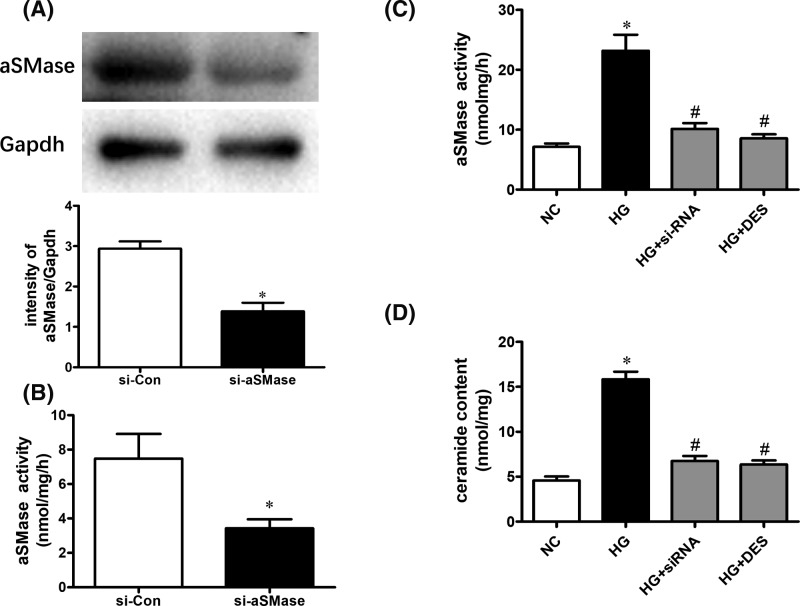
Inhibition of aSMase activity decrease ceramide production in endothelial cells (**A**) aSMase expression and activity (**B**) was suppressed significantly by transfecting aSMase siRNA. Elevated aSMase activity (**C**) and ceramide content (**D**) induced by HG was suppressed by desipramine treatment and transfecting aSMase siRNA. Data are shown as the mean ± S.E.M. with *n*=4–5 per group. ^*^P<0.05 compared with the NC group. ^#^*P*<0.05 compared with the HG group.

**Figure 6:**
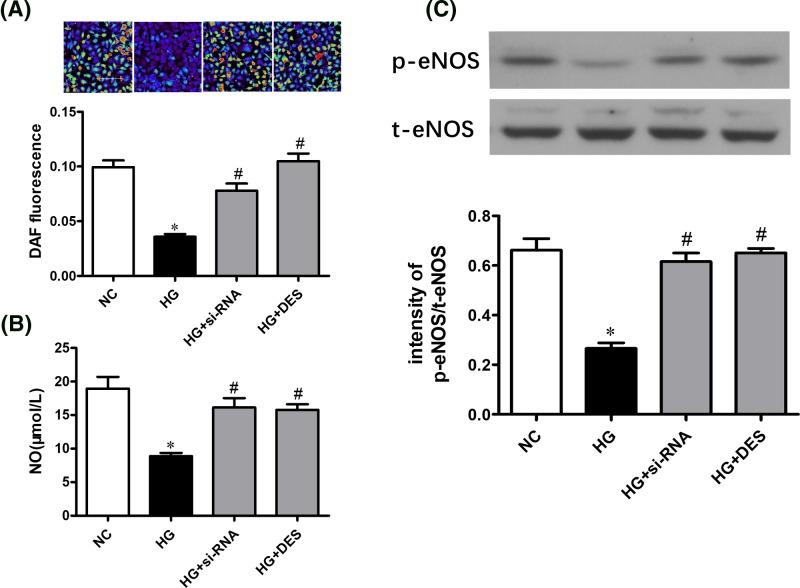
Inhibition of aSMase activity reverses impaired eNOS/NO pathway induced by HG in endothelial cells (**A**) NO generation was measured by DAF-FM-DA fluorescence under various treatments. (**B**) Reduced NO generation induced by HG was restored by transfecting aSMase siRNA or desipramine treatment in endothelial cells. (**C**) Impaired eNOS phosphorylation induced by HG was improved by transfecting aSMase siRNA or desipramine treatment in endothelial cells. Data are shown as the mean ± S.E.M. with *n*=4–5 per group. ^*^P<0.05 compared with the NC group. ^#^*P*<0.05 compared with the HG group.

To further determine the role of aSMase in endothelial dysfunction induced by HG, we knocked down aSMase expression by transfecting siRNA. As expected, aSMase siRNA successfully suppressed protein expression (Figure 5A) and aSMase activity (Figure 5B). The aSMase activity and ceramide content increased significantly in endothelial cells with HG and were restored by using si-RNA or desipramine treatment (Figure 5C,D). HG decreased NO production in endothelial cells, as indicated by the DAF-FM diacetate fluorescence intensity, while NO reduction was restored by treatment with si-RNA or desipramine (Figure 6A,B). In addition, inhibition of aSMase/ceramide pathway reversed the reduced eNOS phosphorylation induced by high-glucose in endothelial cells (Figure 6C).

## Discussion

Our findings extend our previous research in which we reported that the negative effects of HG on the Akt/eNOS/NO signaling pathway could be improved by inhibiting ceramide synthesis *in vitro* [19]. Here, we demonstrated that these changes can be recapitulated in animal study. The present study determined that treatment with the aSMase inhibitor, desipramine, prevented ceramide accumulation and ameliorated glycemia control and insulin resistance in diabetic animals. These findings were consistent with prior studies [8,9,20]. In addition, we showed that the aSMase inhibitor attenuated ceramide levels and improved vascular function in diabetic animals.

Inhibition of ceramide accumulation could improve glucose intolerance and blood glucose levels in diabetic animals [6,21–23]. In addition, reducing ceramide biosynthesis could improve endothelial dysfunction in diabetic animals by reducing metabolic abnormalities, which have been shown to induce vascular dysfunction [6,12]. Ceramide is an important signaling molecule and locate in the center of sphingolipid metabolism. Biologically active ceramide is mainly generated from sphingomyelin by the activation of sphingomyelinase. Acid and neutral sphingomyelinases ubiquitously expressed in most tissues [24], and endothelial cells are the main source of aSMase [25–27]. Elevated aSMase activity was observed in patients and animals with diabetes [8,9, 28]. Moreover, high-fat-fed ldlr^–/–^ mice displayed insulin resistance and high blood glucose, while in ldlr^–/–^ mice lacking aSMase, these metabolic abnormalities could be prevented [29]. Activation of aSMase can induce a higher transformation rate of sphingomyelin-ceramide and leads to endothelial dysfunction in diabetic mice [30]. To date, most studies have explored the relationship between ceramide and diabetes by regulating the *de novo* biosynthesis pathway. In the present study, we detected whether inhibiting aSMase activity could negate ceramide accumulation and endothelial dysfunction in diabetes.

Here, we demonstrated that aSMase/ceramide pathway plays an important role in diabetes-related vascular dysfunction. Chronic treatment with aSMase inhibitor could reverse the impaired endothelium-dependent vascular relaxation induced by diabetes. Consistently, we also found that impaired NO production was improved by desipramine treatment. The expression of phospho-eNOS-Ser1177 was suppressed in arteries from diabetic animals, which was consistent with previous investigations [6,31]. Additionally, desipramine treatment increased the expression of phospho-eNOS-Ser1177 in arteries from diabetic mice. Thus, the aSMase/ceramide pathway may mediate vascular dysfunction by reglulating eNOS/NO pathway.

The role of aSMase/ceramide pathway in the pathogenesis of diabetic vascular complications is not fully understood [2]. We explored the role of aSMase/ceramide pathway in vascular dysfunction in our studies [6,19]. Our data suggested that aSMase/ceramide accumulation impaired eNOS/NO pathway and endothelial dysfunction in HG condition or diabetes. In conclusion, our results suggest that inhibition of aSMase/ceramide pathway improves endothelium-dependent vasodilation in diabetic animals. Therefore, aSMase/ceramide pathway may be a potential target for the treatment of vascular dysfunction in diabetes mellitus.

## Abbreviations

aSMase: acid sphingomyelinase
DC: diabetes control
eNOS: endothelial NO synthase
H&E: hematoxylin and eosin
HG: high glucose
LG: L-glucose
NC: normal control
NO: nitric oxide
NPSS: normal physiological saline solution
si-aSMase: specific aSMase
UPLC: ultra performance liquid chromatography

## References

[1] Chaurasia B. and Summers S.A. (2015) Ceramides - lipotoxic inducers of metabolic disorders. Trends Endocrinol. Metab. 26, 538–550 10.1016/j.tem.2015.07.006 26412155

[2] Galadari S., Rahman A., Pallichankandy S., Galadari A. and Thayyullathil F. (2013) Role of ceramide in diabetes mellitus: evidence and mechanisms. Lipids Health Dis. 12, 98 10.1186/1476-511X-12-98 23835113 PMC3716967

[3] Holland W.L., Brozinick J.T., Wang L.P., Hawkins E.D., Sargent K.M., Liu Y. (2007) Inhibition of ceramide synthesis ameliorates glucocorticoid-, saturated-fat-, and obesity-induced insulin resistance. Cell Metab. 5, 167–179 10.1016/j.cmet.2007.01.002 17339025

[4] Yang G., Badeanlou L., Bielawski J., Roberts A.J., Hannun Y.A. and Samad F. (2009) Central role of ceramide biosynthesis in body weight regulation, energy metabolism, and the metabolic syndrome. Am. J. Physiol. Endocrinol. Metab. 297, E211–24 10.1152/ajpendo.91014.2008 19435851 PMC2711669

[5] Frangioudakis G., Garrard J., Raddatz K., Nadler J.L., Mitchell T.W. and Schmitz-Peiffer C. (2010) Saturated- and n-6 polyunsaturated-fat diets each induce ceramide accumulation in mouse skeletal muscle: reversal and improvement of glucose tolerance by lipid metabolism inhibitors. Endocrinology 151, 4187–4196 10.1210/en.2010-0250 20660065 PMC2940499

[6] Chun L., Junlin Z., Aimin W., Niansheng L., Benmei C. and Minxiang L. (2011) Inhibition of ceramide synthesis reverses endothelial dysfunction and atherosclerosis in streptozotocin-induced diabetic rats. Diabetes Res. Clin. Pract. 93, 77–85 10.1016/j.diabres.2011.03.017 21492950

[7] Meikle P.J. and Summers S.A. (2017) Sphingolipids and phospholipids in insulin resistance and related metabolic disorders. Nat. Rev. Endocrinol. 13, 79–91 10.1038/nrendo.2016.169 27767036

[8] Górska M., Barańczuk E. and Dobrzyń A. (2003) Secretory Zn2+-dependent sphingomyelinase activity in the serum of patients with type 2 diabetes is elevated. Horm. Metab. Res. 35, 506–507 10.1055/s-2003-41810 12953170

[9] Opreanu M., Tikhonenko M., Bozack S., Lydic T.A., Reid G.E., McSorley K.M. (2011) The unconventional role of acid sphingomyelinase in regulation of retinal microangiopathy in diabetic human and animal models. Diabetes 60, 2370–2378 10.2337/db10-0550 21771974 PMC3161322

[10] Kady N., Yan Y., Salazar T., Wang Q., Chakravarthy H., Huang C. (2017) Increase in acid sphingomyelinase level in human retinal endothelial cells and CD34+ circulating angiogenic cells isolated from diabetic individuals is associated with dysfunctional retinal vasculature and vascular repair process in diabetes. J. Clin. Lipidol. 11, 694–703 10.1016/j.jacl.2017.03.007 28457994 PMC5492962

[11] Jing W., Minxiang L., Lan L. and Shan W. (2007) Effects of rosiglitazone on endothelium - dependent vasodilation function of aorta in type 2 diabetic rats. Chin. J. Diab. 15, 621–624

[12] Zhang Q.J., Holland W.L., Wilson L., Tanner J.M., Kearns D., Cahoon J.M. (2012) Ceramide mediates vascular dysfunction in diet-induced obesity by PP2A-mediated dephosphorylation of the eNOS-Akt complex. Diabetes 61, 1848–1859 10.2337/db11-1399 22586587 PMC3379648

[13] Bharath L.P., Ruan T., Li Y., Ravindran A., Wan X., Nhan J.K. (2015) Ceramide-initiated protein phosphatase 2A activation contributes to arterial dysfunction *in vivo*. Diabetes 64, 3914–3926 10.2337/db15-0244 26253611 PMC4613970

[14] Luo Y. and Lei M. (2017) α-Mangostin protects against high-glucose induced apoptosis of human umbilical vein endothelial cells. Biosci. Rep. 37, 10.1042/BSR20170779 PMC5725610 29054969

[15] He X., Chen F., Dagan A., Gatt S. and Schuchman E.H. (2003) A fluorescence-based, high-performance liquid chromatographic assay to determine acid sphingomyelinase activity and diagnose types A and B Niemann-Pick disease. Anal. Biochem. 314, 116–120 10.1016/S0003-2697(02)00629-2 12633609

[16] He X., Huang Y., Li B., Gong C.X. and Schuchman E.H. (2010) Deregulation of sphingolipid metabolism in Alzheimer’s disease. Neurobiol. Aging 31, 398–408 10.1016/j.neurobiolaging.2008.05.010 18547682 PMC2829762

[17] Lau Y.S., Mustafa M.R., Choy K.W., SMH C., Potocnik S., Herbert T.P. (2018) 3′,4′-dihydroxyflavonol ameliorates endoplasmic reticulum stress-induced apoptosis and endothelial dysfunction in mice. Sci. Rep. 8, 1818 10.1038/s41598-018-19584-8 29379034 PMC5789000

[18] Lau Y.S., Tian X.Y., Huang Y., Murugan D., Achike F.I. and Mustafa M.R. (2013) Boldine protects endothelial function in hyperglycemia-induced oxidative stress through an antioxidant mechanism. Biochem. Pharmacol. 85, 367–375 10.1016/j.bcp.2012.11.010 23178655

[19] Wang A., Li C., Liao J., Dong M., Xiao Z. and Lei M. (2013) Ceramide mediates inhibition of the Akt/eNOS pathway by high levels of glucose in human vascular endothelial cells. J. Pediatr. Endocrinol. Metab. 26, 31–38 10.1515/jpem-2012-0144 23457308

[20] Kobayashi Keiko, *. I.I., Nakagawa Tomoka, K. C., Kitamura Yuko, K. E. (2011) Increase in plasma ceramide levels via secretory sphingomyelinase activity in streptozotocin-induced diabetic rats. Med. Chem. Commun. 2, 536–541 10.1039/c0md00154f

[21] Ussher J.R., Koves T.R., Cadete V.J., Zhang L., Jaswal J.S., Swyrd S.J. (2010) Inhibition of *de novo* ceramide synthesis reverses diet-induced insulin resistance and enhances whole-body oxygen consumption. Diabetes 59, 2453–2464 10.2337/db09-1293 20522596 PMC3279530

[22] Li Z., Zhang H., Liu J., Liang C.P., Li Y., Li Y. (2011) Reducing plasma membrane sphingomyelin increases insulin sensitivity. Mol. Cell. Biol. 31, 4205–4218 10.1128/MCB.05893-11 21844222 PMC3187286

[23] Kurek K., Mikłosz A., Łukaszuk B., Chabowski A., Górski J. and Żendzian-Piotrowska M. (2015) Inhibition of ceramide *De Novo* synthesis ameliorates diet induced skeletal muscles insulin resistance. J. Diabetes Res. 2015, 154762 10.1155/2015/154762 26380311 PMC4562089

[24] Busik J.V., Esselman W.J. and Reid G.E. (2012) Examining the role of lipid mediators in diabetic retinopathy. Clin. Lipidol. 7, 661–675 10.2217/clp.12.68 23646066 PMC3640872

[25] Marathe S., Schissel S.L., Yellin M.J., Beatini N., Mintzer R., Williams K.J. (1998) Human vascular endothelial cells are a rich and regulatable source of secretory sphingomyelinase. Implications for early atherogenesis and ceramide-mediated cell signaling. J. Biol. Chem. 273, 4081–4088 10.1074/jbc.273.7.4081 9461601

[26] Andrieu-Abadie N. and Levade T. (2002) Sphingomyelin hydrolysis during apoptosis. Biochim. Biophys. Acta 1585, 126–134 10.1016/S1388-1981(02)00332-3 12531545

[27] Opreanu M., Lydic T.A., Reid G.E., McSorley K.M., Esselman W.J. and Busik J.V. (2010) Inhibition of cytokine signaling in human retinal endothelial cells through downregulation of sphingomyelinases by docosahexaenoic acid. Invest. Ophthalmol. Vis. Sci. 51, 3253–3263 10.1167/iovs.09-4731 20071681 PMC2891477

[28] Kady N., Yan Y., Salazar T., Wang Q., Chakravarthy H., Huang C. (2017) Increase in acid sphingomyelinase level in human retinal endothelial cells and CD34(+) circulating angiogenic cells isolated from diabetic individuals is associated with dysfunctional retinal vasculature and vascular repair process in diabetes. J. Clin. Lipidol. 11, 694–703 10.1016/j.jacl.2017.03.007 28457994 PMC5492962

[29] Deevska G.M., Rozenova K.A., Giltiay N.V., Chambers M.A., White J., Boyanovsky B.B. (2009) Acid sphingomyelinase deficiency prevents diet-induced hepatic triacylglycerol accumulation and hyperglycemia in mice. J. Biol. Chem. 284, 8359–8368 10.1074/jbc.M807800200 19074137 PMC2659194

[30] Chakravarthy H., Navitskaya S., O’Reilly S., Gallimore J., Mize H., Beli E. (2016) Role of acid sphingomyelinase in shifting the balance between proinflammatory and reparative bone marrow cells in diabetic retinopathy. Stem Cells 34, 972–983 10.1002/stem.2259 26676316 PMC5088619

[31] Li Q., Atochin D., Kashiwagi S., Earle J., Wang A., Mandeville E. (2013) Deficient eNOS phosphorylation is a mechanism for diabetic vascular dysfunction contributing to increased stroke size. Stroke 44, 3183–3188 10.1161/STROKEAHA.113.002073 23988642 PMC3864831

